# Bridging Policy and Practice: Applicability of Brazil’s Healthy Lifestyle Promotion Model in the Philippine Context

**DOI:** 10.34172/hpp.45279

**Published:** 2026-06-06

**Authors:** Mary Sandie Jaymie Mahusay Tan

**Affiliations:** University of Eastern Philippines, Associate Professor, College of Medicine, University Town Northern Samar 6400, Philippines

## To the Editor,

 I read with great enthusiasm the article “Implementation of public health policies for healthy lifestyles promotion: what Brazil should tell us?” by Ferrari,^1^ published in Health Promotion Perspectives. The paper offers valuable insights for countries such as the Philippines, which are navigating similar epidemiologic and socioeconomic transitions. Like Brazil, the Philippines faces a dual burden of disease, where noncommunicable diseases (NCDs) such as hypertension, diabetes, and cardiovascular diseases continue to increase alongside persistent infectious diseases. Although the national government has developed several programs to address these concerns, issues of sustained implementation, multi-sectoral coordination, and cultural alignment as emphasized by Ferrari remain under-addressed in the local context.

 In the Philippines, cultural practices such as fiestas, communal eating (salu-salo), and prevailing perceptions of body image strongly shape dietary habits and physical activity behavior. Existing studies support this observation. For example, Pengpid et al.^2^ found that seven in ten Filipino adolescents skipped one or more meals within a month, with meal skipping linked to poorer dietary and mental health outcomes. Filipino adults exhibit high rates of obesity and hypertension, influenced by high-sodium diets and frequent consumption of refined carbohydrates.^3^ These findings suggest that public health policies must account for sociocultural determinants to be effective.

 Moreover, a national survey on dietary behavior reported low awareness of food-based dietary guidelines wherein only 10% of household meal planners recognized the Pinggang Pinoy® framework which is an inconsistent adherence even among those aware.^4^ This gap between awareness and behavioral adoption illustrates that while health messages exist, they often fail to resonate culturally or translate into practice.

 In the Philippine setting, studies seldom explore how health promotion programs reach marginalized populations. Issues of equity, accessibility, and inclusion remain critical to ensuring that policies reach those who need them most. For instance, local studies show that socioeconomic status influences NCD prevention behaviors such as socioeconomic status influences NCD prevention behaviors; for example, lower-income households demonstrate less access to preventive care and healthy diets.^3^ Likewise, physical activity research among university students revealed barriers such as lack of time, inadequate facilities, and low motivation, with greater challenges experienced by students from public institutions. ^5^

 At the implementation level, national directives frequently fail to translate effectively at the barangay (village) level. Fragmented governance, resource disparities, and weak community engagement hinder policy continuity. Implementation research is thus essential to evaluate how national policies are contextualized locally. Campaigns that frame health as a shared responsibility rather than an individual burden align better with Filipino collectivist values and could improve participation and sustainability.^6^

 Ferrari’s analysis provides a meaningful foundation for countries like the Philippines to re-evaluate and strengthen health promotion frameworks. The paper highlights that cultural adaptation, intersectoral collaboration, and monitoring should operate in synergy for policies to be impactful. By addressing policy gaps through strong collaboration, culturally responsive design, and evidence-based policymaking, any nation can evolve toward a more inclusive and sustainable model for lifestyle promotion.

 The challenges faced by the Philippines in addressing NCDs mirror those documented by Ferrari (2018) for Brazil. While policy frameworks and health campaigns are in place, their success depends on translating national intent into culturally resonant and community-driven action. Through culturally adaptive, evidence-informed, and equity-oriented strategies, the Philippines can bridge the gap between policy and practice and move toward a sustainable model for health promotion.

## Competing Interests

 The authors declare that they have no financial, personal, or professional relationships that could be perceived as influencing the objectivity or integrity of the work described in this letter.

## Ethical Approval

 The authors declare that they have no financial, personal, or professional relationships that could be perceived as influencing the objectivity or integrity of the work described in this letter.
